# Making and breaking of chemical bonds in single nanoconfined molecules

**DOI:** 10.1126/sciadv.abq7776

**Published:** 2022-09-09

**Authors:** Ole Bunjes, Daniel Hedman, Alexandra Rittmeier, Lucas A. Paul, Inke Siewert, Feng Ding, Martin Wenderoth

**Affiliations:** ^1^ IV. Physikalisches Institut, Georg-August-Universität Göttingen, Friedrich-Hund-Platz 1, 37077 Göttingen, Germany.; ^2^ Center for Multidimensional Carbon Materials, Institute for Basic Science (IBS), Ulsan 44919, Republic of Korea.; ^3^ Institut für Anorganische Chemie, Georg-August-Universität Göttingen, Tammannstraße 4, 37077 Göttingen, Germany.; ^4^ Department of Materials Science and Engineering, Ulsan National Institute of Science and Technology (UNIST), Ulsan 44919, Republic of Korea.

## Abstract

Nanoconfinement of catalytically active molecules is a powerful strategy to control their chemical activity; however, the atomic-scale mechanisms are challenging to identify. In the present study, the site-specific reactivity of a model rhenium catalyst is studied on the subnanometer scale for complexes confined within quasi–one-dimensional molecular chains on the Ag(001) surface by scanning tunneling microscopy. Injection of tunneling electrons causes ligand dissociation in single molecules. Unexpectedly, while half of the complexes show only the dissociation, the confined molecules show also the reverse reaction. On the basis of density functional theory calculations, this drastic difference can be attributed to the limited space in confinement. That is, the split-off ligand adsorbs closer to the molecule and the dissociation causes less structural disruption. Both of these facilitate the reverse reaction. We demonstrate formation and disruption of single chemical bonds of nanoconfined molecules with potential application in molecular data storage.

## Teaser

Confinement of molecules yields reversible dissociation by limitation of adsorption sites and by structure stabilization.

## INTRODUCTION

One major challenge of our time is to cope with climate change (*1*), and converting CO_2_ into valuable products is a strategy to minimize the net CO_2_ emissions (*2*, *3*). In this context, the development of catalytically active molecular complexes and of functional molecule-surface hybrids is one focus of research (*4*–*7*).

Fundamentally, after a full catalytic cycle, the catalyst returns to its original state allowing for the consecutive reaction, with this intrinsically relying on microscopic reversibility.

For the realization of on-surface atomic-scale memory with highest data storage densities, diverse reversible mechanisms have been suggested, e.g., the manipulation of nanoscopic magnets (*8*), the atomic-scale precise shifting of vacancies (*9*), the stabilization of excited states (*10*), and isomerization reactions of molecules (*11*). To transfer the functionality of a molecular catalyst to this field, it is necessary that first, the complexes can be excited with atomic-scale precision and second, the triggered reactions follow very well-controlled reaction pathways.

Scanning tunneling microscopy (STM) allows to control chemical reactions of individual molecules confined to surfaces with utmost precision, which enables, e.g., the investigation of charged intermediate states (*12*) and of highly reactive molecular species (*13*). STM enables creating and breaking single chemical bonds, e.g., for ligand transfer (*14*), and provides diverse reaction control parameters for spectroscopic and real-space studies: (i) the injected tunneling electrons, (ii) their energy, (iii) the electric field in the tunneling junction, and (iv) the proximity of the STM tip (*15*–*19*). With this, STM can contribute to the understanding of single-molecule reactions that are of fundamental interest (*20*), even under otherwise hardly accessible nanoconfinement conditions (*19*, *21*).

In this work, we make use of the self-assembly of the model CO_2_-reduction catalyst *fac*-Re(bpy)(CO)_3_Cl (bpy = 2,2′-bipyridine) on the Ag(001) surface that results in a nanoconfinement of the molecular complexes within quasi–one-dimensional molecular chains. Because of its catalytic activity and an in-depth mechanistic understanding (*22*, *23*), the rhenium catalyst still acts as model complex for the development of new catalytic complexes and hybrid systems (*5*, *7*, *24*, *25*). We use nanoconfinement, which is a powerful strategy for pushing molecular catalysts to their highest performances (*26*) and has attracted considerable interest because it is known to alter the chemical properties of confined entities, for affecting their mobility, and for selectively restricting the reaction parameter space (*19*–*21*, *27*–*31*).

The reactions studied are the bond-breaking (dissociation) and the bond-formation (reattachment) reactions of the chloride ligand from and to the metal center of the complex. Ligand dissociation is the initial step of the catalytic cycle, which is here triggered by local injection of tunneling electrons. In particular, the complexes align in two different ways on the surface inducing two drastically differing characteristic responses to the electronic excitation. The complexes at the border of the blocks (labeled border molecules) do only show the dissociation reaction, but unexpectedly, the complexes confined within the molecular blocks (labeled center molecules) show both forward and reverse reactions.

In this way, the self-assembled molecular structures provide a robust local environment that allows to directly contrast the reactivity of two representatives of the very same molecular species. We demonstrate the impact of the molecular self-confinement on an electronically induced single-bond-splitting chemical reaction on a mechanistic level. The studied system may be a candidate to be used for information storage where the formation and breaking of single chemical bonds can function as both permanent and rewritable memory on the atomic scale. Appealingly, the self-assembly approach makes upscaling feasible (*32*).

## RESULTS

### Molecular monolayers on Ag(001)

**Fig. 1.**
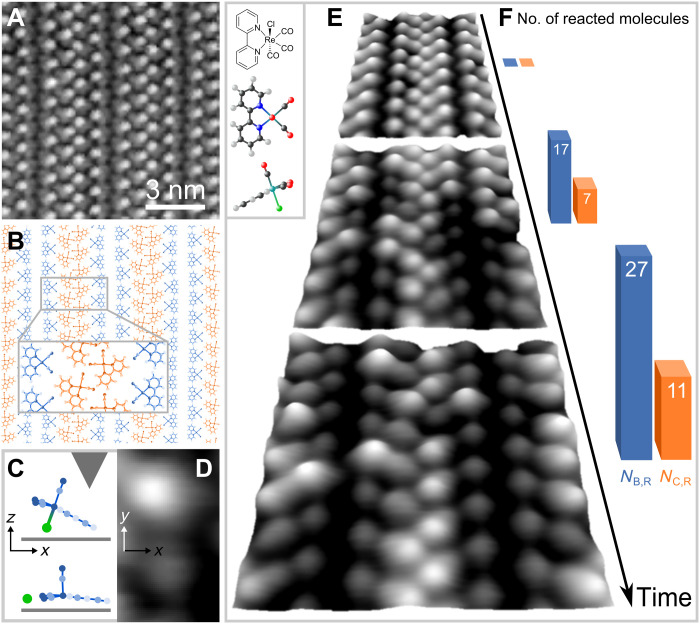
Reacting nanoconfined molecules on Ag(001). (**A**) Topography (*U*_bias_ = 1 V, *I*_set_ = 50 pA) of molecular monolayer. (**B**) Structural model (top view) showing pattern of molecules forming border (blue) and center (orange) of nanoconfinement. Inset: Molecular building block. (**C**) Structural model of intact and dissociated molecular complex on silver underneath the STM tip (side view). Chloride ion depicted in green. (**D**) Topography (*U*_bias_ = 2 V, *I*_set_ = 50 pA, 1.2 nm by 2 nm) of neighboring border molecules. Upper molecule in original state *O*, lower molecule has reacted to state *R*, corresponding to (C). (**E**) Typical time evolution of a monolayer section (7.4 nm by 7.4 nm) during excitation scan at *U*_bias_ = 2 V and *I*_set_ = 50 pA. Top: Unreacted monolayer as reference, *t* = 0 hours. Middle: Monolayer at *t* ~ 5 hours. Bottom: Same area as center at *t* ~ 9.5 hours. Times determined as in Fig. 2A. Inset: Structural formula of *fac*-Re(bpy)(CO)_3_Cl (bpy = 2,2′-bipyridine) and ball-and-stick models (top and side view). Colors: Gray, C; white, H; red, O; blue, N; green, Cl; turquoise, Re atom. Number of reacted molecules *N*_B/C,R_ shown as bar plot in (**F**).

We prepared monolayers of the rhenium complex *fac*-Re(bpy)(CO)_3_Cl (bpy = 2,2′-bipyridine), the structure of which is depicted in the inset of Fig. 1E, on a clean Ag(001) surface by sublimation. Imaging these layers using STM with sufficiently low bias voltages (imaging mode) leaves the molecules unaffected, and they remain in their original as deposited state, which we call state *O*. A typical topography of the monolayer, shown in Fig. 1A, demonstrates the high quality of the long-range ordered self-assembled molecular pattern. The alignment of the molecules on the surface has recently been clarified (*33*). The energetically most favorable configuration is with the Cl ligand facing the surface, as depicted in the side view in Fig. 1C. The structural arrangement of the complexes inside the monolayer of Fig. 1A can be found in Fig. 1B. The monolayer is built up of molecular blocks each of which consists of four columns of molecules (Fig. 1B, inset). From left to right, there are uncovered silver atoms (not shown) followed by one column of border molecules (blue) of which the bipyridine is aligned in parallel to the [110] crystal direction of the substrate. Next, there are two columns of confined center molecules (orange) for which there is an angle of about ±45° between bipyridines and [110] direction. The block is terminated by a second column of border molecules (blue) mirroring the orientation of the first, and uncovered silver atoms (not shown) follow before the next block starts.

### Temporal excitation measurements

Scanning a surface area at increased bias voltage (excitation mode) causes a fraction of the molecules to transition into a different reacted state labeled as *R*. The reaction is evident as a change of contrast of the corresponding molecule, as can be seen in Fig. 1D. While the molecule in state *O* (top) appears bright, the molecule in state *R* (bottom) shows a darker contrast. To study the reaction kinetics on the single-molecule level, the molecules’ reaction states are monitored by scanning a fixed surface area in excitation mode repeatedly. The typical temporal evolution is depicted in Fig. 1E. From top to bottom, the three topographies show a monolayer each at a later time during an excitation scan. In the topmost topography, which serves as reference, no molecules are in state *R*, but the number *N*_R_ of molecules in this state increases with time. The knowledge of the molecules’ local arrangement within the pattern allows for a detailed site-specific evaluation of the observed reactions.

One central observation is, quantifying *N*_R_(*t*) for the topographies in Fig. 1E, the excitation of the molecules shows local differences between border (B) and center (C) molecules. As evident in Fig. 1F, for later times, *N*_B,R_ is considerably higher than *N*_C,R_.

**Fig. 2.**
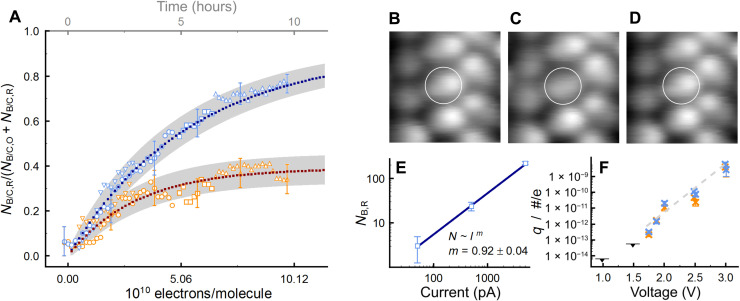
Reaction triggered by one-electron process. (**A**) Normalized *N*_B/C,R_(*t*) (colors: blue, border; orange, center), at constant tunneling parameters of *U*_bias_ = 2 V and *I*_set_ = 50 pA, 10 nm by 10 nm, *t*_scan_ = 572 ± 4 s. Geometric shapes, different datasets; dotted lines, rate equation model; gray shadow, SD; details in Materials and Methods. Effective rate constants: *k*_B,O→R_ = 2.7 ± 0.1 × 10^−3^ s^−1^, *k*_B,R→O_ = 2.8 ± 0.2 × 10^−4^ s^−1^, *k*_C,O→R_ = 1.8 ± 0.1 × 10^−3^ s^−1^, *k*_C,R→O_ = 2.8 ± 0.2 × 10^−3^ s^−1^. Selection of error bars shown for clarity. (**B** to **D**) Topographies (*U*_bias_ = 2 V, *I*_set_ = 50 pA, 2.6 nm by 2.9 nm) of one monolayer region. Encircled molecule in (B) in state *O*, switches to reacted state *R* (C), and back to state *O* (D). Note that reverse reaction can occur if all surrounding molecules are in state *O* (fig. S10). (**E**) *N*_B,R_(*I*_set_) at *U*_bias_ = 2 V, *t*_scan_ = 300 ± 5 s; details in Materials and Methods. Dark blue line, exponential fit; exponent *m* = 0.92 ± 0.04. Scan at *I*_set_ = 5 pA did not induce single reaction and is not depicted. (**F**) Quantum efficiency *q* = #reactions/electron of the induced chemical reaction as function of voltage. Gray, dashed line, guide to the eye; black triangles, upper boundary as no reaction was induced.

The analysis of successive topographies acquired over the course of several hours can be seen in Fig. 2A, upper x axis. At short durations and for small electron doses (lower x axis), both orientations of molecules show similar evolutions with time, i.e., a similar quasi-linear increase of$\frac{N_{B/C,R}}{N_{B/C,R}+N_{B/C,R}}(t)$. Because in this regime the statistical significance for the center molecules is lower, this experiment was repeated using a different tip, a different sample, and a similar bias voltage yielding the same result with less stochastic fluctuations due to the larger number of molecules studied. These data can be found in fig. S1.

For longer durations and higher electron doses, the slope of *N*_B,R_(*t*) is reducing, which can be attributed to the finite size of the studied surface area, while even after 10 hours saturation is not reached. In contrast, the center molecules reach saturation at 30 to 40% after 7 hours. The origin of these obvious differences can be identified on the local scale. The center molecules are found to switch reversibly between both states, shown exemplarily in Fig. 2 (B to D). This observation was confirmed by studying several molecules at various energies and using different tips. In contrast, at the given electron energy of 2 eV and for the investigation conducted at 1.875 eV (cf. fig. S1), not a single reverse reaction was observed for the border molecules during all the scans.

To quantify the observed dynamics, we used a rate equation model for a two-level system allowing for transitions between both states *O* and *R*. This yields the two equations for border (B) and center molecules (C)

$${\overset{\cdot }{N}}_{B/C,R}=k_{B/C,O\to R}(N_{B/C,\text{total}}-N_{B/C,R})-k_{B/C,R\to O}N_{B/C,R}$$ (1)

The effective rate constants *k*_B/C,O→R_ and *k*_B/C,R→O_ were used as parameters to model the experimental data. Details can be found in Materials and Methods. In short, this was used for a simulation on a discrete and finite lattice to estimate the statistical deviation of the data from the analytic solution due to the finite number of molecules and the stochastic nature of the processes studied. As shown in Fig. 2A, the simulated data very well resemble the experimental data.

In total, in excitation mode, 116 forward reactions were found for the border molecules and 110 for the center molecules. The total number of reverse reactions observed for the center molecules at 1.875 and 2 V was 40. During the same period, not a single reverse reaction was observed for the border molecules. The effective rate constant for the reverse reaction of the border molecules is a factor of 10 smaller for the forward reaction rate for the 2 V measurement and several orders of magnitude smaller for the 1.875 V measurement. Furthermore, only a single reverse reaction was found for excitation scans at elevated bias voltages between 3.1 and 3.4 V (cf. fig. S2), while at the same time 55 reverse reactions were found for the center molecules. Thus, we interpret the reverse reaction rate for the border molecules to be negligible.

As a complementary experiment, the current was used as explicit control parameter (see Materials and Methods), the result can be seen in Fig. 2E. For the three orders of magnitude studied, fitting the exponential relation *N*_B,R_ ~ (*I*_set_)*^m^*, with the exponent *m* being the fitting parameter, results in *m* = 0.92 ± 0.04. This close to linear dependence is characteristic for a one-electron process (*15*). In this picture, the quantum efficiency *q*, the number of induced reactions per electron, can be studied as a function of the electron energy, as shown in Fig. 2F. While for 0 V < *U*_bias_ ≤ 1.5 V no reactions were observed, up to 3 V the efficiency *q* is found to increase by four orders of magnitude.

### Pulsed experiments

**Fig. 3.**
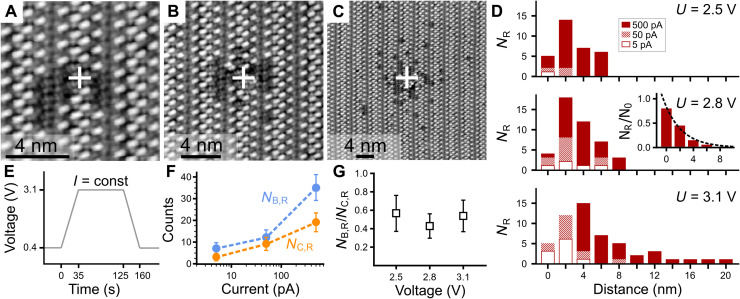
Reactions induced nonlocally. (**A**) Topography after voltage pulse (E) at *I*_set_ = 5 pA has been applied at position of white cross. Darker molecules have switched into state *R*. (**B**) After second pulse at *I*_set_ = 50 pA. (**C**) After third pulse at *I*_set_ = 500 pA. *U*_bias_ = 0.4 V, *I*_set_ = 50 pA. (**D**) Histograms of reacted molecules as function of distance from electron injection site. For different voltages, previously unreacted monolayer regions were used. Inset: Normalization to number of available molecules; details in Materials and Methods. Dashed line, exponential fit; lateral decay constant λ_2.8V_ = 2.7 ± 0.2 nm^−1^. For 3.1 V, bars for 0 and 2 nm distance are not evaluated as here secondary reactions occur that are beyond the scope of the current work. (**E**) Pulse scheme for the experiments carried out at *U*_bias_ = 3.1 V keeping the current constant. The same scheme was applied for all different voltages. (**F**) *N*_R_(*I*_set_) for the *U*_bias_ = 3.1 V-pulses evaluated separately for both orientations of the molecules. Dashed lines, guides to the eye. (**G**) Ratio *N*_C,R_/*N*_B,R_ (*I*_set_ = 500 pA) at different pulse heights but at a constant number of electrons.

To characterize the local distribution of induced reactions, experiments were performed applying voltage pulses, as depicted in Fig. 3E, with the tip being at a laterally fixed position and keeping the tunneling current constant. After a pulse, the corresponding surface area was scanned in imaging mode. Pulses at different currents were applied consecutively in ascending order within the same surface area and for different voltages each time a previously undisturbed monolayer was used (further details in Materials and Methods). Figure 3 (A to C) shows the resulting spatial distributions after pulses at *U*_bias_ = 3.1 V with currents of *I*_set_ = 5, 50, and 500 pA. While the outermost molecules remain unaffected, several of the molecules in the center of the image switched into state *R*. With increasing current, the number of molecules in state *R* increases, and the distribution is found to broaden (cf. Fig. 3, A to D). Qualitatively similar behaviors are found for varying pulse heights, as evident by the histograms shown in Fig. 3D. The distribution shows broadening for the same currents at higher voltages, but most notably, both the lateral width of the area in which molecules are reacted and the absolute number of molecules increase with higher currents. This shows that the reaction is induced nonlocally and underlines the reliance of the reaction on the number of injected electrons. Normalization to the number of molecules within the corresponding annulus (details in Materials and Methods), shows a rapid decay for *N*_R_(*r*) with increasing distance *r* from the electron injection site, as exemplarily shown in the inset of Fig. 3D. The exponential fit yields an average lateral decay constant of λ = 2.1 ± 0.2 nm^−1^ considering all three distributions at the highest current.

Evaluation of the spatial distributions separately for both orientations of molecules is shown in Fig. 3F for a pulse height of 3.1 V. With increasing current, *N*_B/C,R_ is found to increase. For the largest current, *N*_B,R_ > *N*_C,R_, which holds for all bias voltages (cf. Fig. 3G) resulting in an averaged ratio$\frac{N_{C,R}}{N_{B,R}}=0.5\pm 0.1$ considering the results from the pulses at the highest current. Besides, there is a tendency to find a smaller ratio closer to the electron injection site and a ratio close to one for increased distances. Averaging over all three distributions at the highest current leads to a ratio of $\frac{N_{C,R}}{N_{B,R}}(r\leq r_{75\%})=0.4\pm 0.1$ within the radius *r*_75%_ in which 75% of the reacted molecules are found and a ratio of $\frac{N_{C,R}}{N_{B,R}}(r>r_{75\%})=0.8\pm 0.3$ at longer distances.

### Comparing border and center molecules

Over a relatively large energy range, we find very similar reaction rates for both orientations (cf. Fig. 2F). This suggests, according to the Bell-Evans-Polanyi principle (*34*, *35*), that the induced reactions are the same for both orientations of the molecule. This fits that we found a single reverse reaction of a border molecule at elevated bias voltages >3 V. Although the reverse reaction rate is much smaller than for the center molecules, this shows that the reverse reaction is principally also possible for the border molecules, supporting the conclusion that for both orientations, the same reaction is induced.

The reaction of the border molecules is found to depend linearly on the tunneling current and is caused by a one-electron process (cf. Fig. 2E); likewise, the reaction of the center molecules is induced by a one-electron process. This is supported by the facts that, first, the reactions for both orientations are found to strongly depend on the availability of injected charges (cf. Fig. 3F), and second, both show a very similar behavior in the temporal and in the spatial measurements. The number of available charge carriers has been found to decrease exponentially with increasing distance from the injection site when injected into a metal surface from an STM tip (*16*). This fits the observed exponential decay shown in the inset of Fig. 3D and allows us to interpret the ratio $\frac{N_{C,R}}{N_{B,R}}$ in terms of the provided electron dose, as indicated by the lower *x* axis in Fig. 2A. For short times (cf. Fig. 2A and fig. S1) and for long distances (cf. Fig. 3D), the electron doses are small resulting in a ratio of $\frac{N_{C,R}}{N_{B,R}}\approx 1$. For long times and for short distances, in contrast, the electron doses are high and result in a ratio of $\frac{N_{C,R}}{N_{B,R}}\approx 0.4$. In this way, the pulse experiments spatially resemble the temporal measurements (cf. fig. S3). Moreover, the similar reaction rates for different energies and the ratio close to one for small electron doses show that the reactions of both orientations are independent of each other. The nonlocal nature of the excitation, as evident in Fig. 3, is consequently attributed to scattering of highly energetic charge carriers in the molecule-surface system.

Besides, the reaction was not observed for experiments at close-to-contact distances between tip and molecules (“mechanical contact” achieved by small bias voltages and large currents; cf. fig. S4), which has been shown to be capable of strongly altering the course of a chemical reaction (*18*, *19*). In the studied regime with *U*_bias_ ≥ 1 V, contributions due to mechanical tip-sample contact can be excluded.

Reconsidering that the ratios $\frac{N_{C,R}}{N_{B,R}}$ in the pulsed experiments well resemble the character of the temporal measurements, there is no hint that the reverse reaction depends on the applied bias voltage in a different manner than the forward reaction. It is found that the forward reaction rates depend on the applied bias voltage (cf. Figs. 2F and 3). If, for example, the reverse reaction was exclusively triggered by the applied electric field, then we would expect a different bias voltage dependence than in the forward reaction as the mechanism is different, i.e., the ratio$\frac{N_{C,R}}{N_{B,R}}$ (cf. Fig. 3G), would depend on the applied field, which is not observed. Hence, the reverse reaction is likely to be electron-induced too. The data suggest that the ratio between the quantum efficiencies of the forward and reverse reactions does not depend on the electron energy (comparing the results at high electron doses for 2 eV with the results at around 3 eV). That is why we consider it improbable that the different characteristic of the reverse reaction found for border and center molecules is caused by a difference in the local density of states after dissociation, as this would likely be energy dependent.

### The reaction mechanism

Concerning the final state *R* of the induced reaction, the straightforward electron-induced process is the charging of a molecule. Nonetheless, though may be necessary as intermediate, we can fully exclude this to be the final state of the reaction, as charged states are known to decay rapidly on noninsulating surfaces (*12*, *18*). A second option is the single-bond breaking dissociation of a CO ligand from the metal center. With the help of density functional theory (DFT) calculations, the energetically favorable alignment of the molecules with respect to the surface is with the Cl ligand facing the surface, i.e., one CO group is facing the tip and the other two are aligned in parallel to the surface (*33*). If one CO was split off, then this would likely attach at some random position (on tip or sample) or desorb into vacuum, which stands in direct contrast to the experimental observation of a reversible reaction and the electrochemical findings on the complex in solution (*36*). This option can therefore be excluded. In contrast, as the Cl ligand is facing the silver surface, after dissociation it likely binds to the surface close to the molecule with this in principle, allowing for the reverse reaction. In solution, a one-electron-reduction–triggered chloride-ligand dissociation is known (*37*), which would fit to the observed reliance on injected electrons. A second option could be an inelastic tunneling process triggering the dissociation, also compatible with a one-electron process. Because reversible isomerization reactions can also be electron-induced (*38*), we also considered *fac-* to *mer*-isomerization as a reaction pathway. However, this was reported to require optical excitation with a wavelength of 313 nm [photon energy ~4 eV; (*39*)]. Furthermore, the complex and its derivatives have been characterized electrochemically very profoundly because of their activity in the electrocatalytic CO_2_ reduction (*4*) and electron-induced isomerization has not been reported. Thus, we exclude isomerization as a most plausible reaction upon electron injection and assume electron-induced dissociation and reattachment of the Cl ligand as follow-up reaction, as depicted in Fig. 1C.

### Nudged elastic band calculations of the chloride ion dissociation

**Fig. 4.**
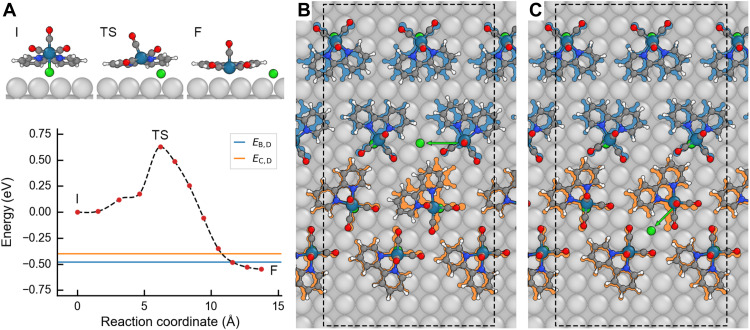
Atomistic modeling of chloride-ion dissociation from the *fac*-Re(bpy)(CO)_3_Cl (bpy = 2,2′-bipyridine) molecule. (**A**) NEB calculations of the chloride-ion-dissociation barrier for a single molecule on Ag(001). Here, red dots denote DFT-calculated energies, the dashed black line is an interpolated spline, and the solid blue and orange lines are the chloride-ion-dissociation energies for the border, *E*_B,D_, and center, *E*_C,D_, molecules, respectively, inside the monolayer (**B** and **C**). The initial (I), transition (TS), and final state (F) of the NEB calculation are also shown in (A). DFT-relaxed geometry for chloride-ion dissociation of (B) one border and (C) one center molecule inside the monolayer. The blue (orange) molecules show the initial relaxed geometry before chloride-ion dissociation of the border (center) molecules, and the green arrows show the dissociation path of the chloride ion (green). The dashed lines in (B) and (C) denote the boundary of the periodic supercell used.

To identify the origin of the two different reaction characteristics observed for border and center molecules, we used extensive DFT-based nudged elastic band (NEB) calculations to study the Cl-ligand dissociation reaction for a single molecule adsorbed on the Ag(001) surface. During the dissociation process, the charge associated with the ligand stays constant, i.e., according to Bader charge analysis of the path depicted in Fig. 4A, it is and stays partially charged (fig. S5). Therefore, the dissociating ligand will be referred to as a chloride ion. The minimum energy pathway for the dissociation with the lowest energy barrier (627 meV) is shown in Fig. 4A. The NEB results for two alternative paths can be found in fig. S6 (A and C). Although the exact shape and the height of the potential barriers differ for the different paths, it is clear that all are too high to be overcome thermally at an experimental temperature of 77 K, i.e., the reaction does not occur spontaneously. This agrees with the experimental findings that the reaction is triggered by electron injection and has never been observed for bias voltages <1 V.

### DFT calculations of the final state

As a second step, we calculated the ground-state energy of the relaxed molecular monolayer built using only intact molecules. This is compared to the ground-state energy of the reacted state obtained by replacing one of the border molecules (Fig. 4B) and center molecules (Fig. 4C) with a reacted molecule and a chlorine add-atom at a corresponding adsorption site and relaxing the structure (which yields a surface-adsorbed chloride ion).

The energy gained by dissociation of one chloride ion, as calculated by Eq. 4, from the border, *E*_B,D_, and center, *E*_C,D_, molecules is shown by the solid lines in Fig. 4A. Here, both configurations (one border molecule reacted versus one center molecule reacted) have similar energies. Consequently, the stability of the final state cannot explain the observed differences in the reactivity. One thing to note is that because of the computational cost of DFT calculations, only a very small model of the molecular monolayer could be used. This will cause the energy penalty for chloride dissociation inside the monolayer to be underestimated because fewer molecules need to rotate/translate. Thus, the dissociation energy for the border and center molecules, *E*_B,D_ and *E*_C,D_, in Fig. 4A, inside the monolayer is likely to be slightly higher.

On the basis of the DFT calculations, we can nonetheless identify differences in the geometry of the relaxed structures before and after dissociation that can explain the differences in the observed reactivity. After dissociation, the chloride ion of the border molecules can relax fully to the third nearest hollow site at a distance of 554 pm, as can be seen in Fig. 4B. This causes substantial distortions in the monolayer. Several molecules show a different adsorption geometry comparing the initial unreacted state to the final reacted state. The biggest distortion is found for the reacted molecule and its nearest center molecule. However, the distortion of the adjacent row of border molecules is also considerable. Rotation/translation of the border molecule, associated with the dissociation, is caused by its slight overlap with a center molecule. After dissociation, the molecule lowers its energy by lying flat on the surface (due to dispersion interactions), which is only possible if the border molecule rotates/translates away from the center molecules.

In contrast, after dissociation, a chloride ion from a center molecule can (due to the limited space available) only relax to the second nearest hollow site (at 390 pm distance), which is much closer to the dissociated molecule, as can be seen in Fig. 4C. Because the center molecules do not overlap with any other molecules in the monolayer, the dissociation of the chloride ion from a center molecule does not cause comparable distortions in the monolayer as evident by Fig. 4C.

Experimentally, for the data recorded in scanning mode, for the transition from state *O* to state *R* the location of the tip at the moment at which the reaction takes place is well known for border molecules (cf. fig. S7). Almost none of the reactions take place while the tip is relatively far away, i.e., there is a sharp contrast change visible in the topography. For the center molecules, on the other hand, for about 20% of the reactions, the tip position is not known, i.e., no sharp contrast change in the STM image due to the reaction. Less structural distortion for a reacting center molecule could contribute to this observation as it would be harder to detect the reaction by distortions of the surrounding molecular structure. Furthermore, comparing high-resolution data of the molecular structure before and after the dissociation reaction has been induced, we find clear evidence that the dissociation of the molecules goes along with structural rearrangements, as is shown in fig. S8. In this way, the experimental observations support the atomistic model.

Now imagine reversing the reaction for border molecules, a long distance has to be overcome by the chloride ion for reattachment and a concerted motion of several neighboring molecules (i.e., many atoms) is necessary to return to the original structure. In contrast, the distance for reattachment at a center molecule is 30% shorter than for the border molecules and less concerted motion of surrounding molecules is necessary for the reattachment of the chloride ion to its center molecule and a reestablishing of the original molecular structure.

## DISCUSSION

At the example of self-assembled molecular monolayers of *fac*-Re(bpy)(CO)_3_Cl (bpy = 2,2′-bipyridine), we demonstrate for identical molecular species in slightly differing local environments two distinct reaction characteristics, which is found to be the consequence of the molecular nanoconfinement. The electron-triggered Cl-ligand dissociation reaction is found to be reversible only for half of the molecules, namely, the ones confined in the center of the molecular building blocks. In sharp contrast, the molecules at the borders of these blocks show negligible rates for the reverse reaction. We identify the origin of this increased barrier for the reverse reaction of the border molecules based on DFT calculations. In comparison to a reacting center molecule, the distance between molecule and dissociated ligand is longer, and secondly, the reaction does go along with substantial disruption of the molecular pattern. Both of these need to be reversed for the ligand-reattachment reaction.

Consequently, to realize a reversible reaction in a molecule-surface hybrid system, e.g., for application in atomic-scale memory, there must be limited space for the dissociated products. Furthermore, the induced reaction may not cause disruption of the surrounding molecular structure. Thereby, on the level of a single molecule, this demonstrates in real space how the chemical reactivity of a complex can be tailored through nanoconfinement. The immobilization of the molecules inside the molecular lattice and the height of the barrier separating initial and final states make the presented system a candidate for realizing atomic-scale data storage at room temperature.

## MATERIALS AND METHODS

### Experimental design

To study the local excitation of nanoconfined molecules, a monolayer-covered silver crystal was prepared by in situ thermal evaporation of *fac*-Re(bpy)(CO)_3_Cl (bpy = 2,2′-bipyridine) onto the clean Ag(001) surface, resulting in the self-assembly of long-range ordered molecular monolayers on the length scale of hundreds of nanometers. Details on the preparation process and on the multifaceted growth behavior of the complex can be found elsewhere (*33*).

### STM on reacted molecular monolayers

All the presented atomic-scale measurements were conducted using a home-built scanning tunneling microscope operating under ultrahigh vacuum conditions (under a pressure of p < 5 × 10^−11^ mbar). For the STM experiments, electrochemically etched tungsten tips were used. The experiments were performed at liquid nitrogen temperature of 77 K. All the data being referred to as topographies were acquired in constant current measuring mode. To correct tilting of the data in *z*-direction planes were fitted to physically flat areas. For better visibility, filters typical for improving the quality of STM topographies, like subtraction of line averages or matrix mean and median filters, were applied making sure not to change the physical content to be interpreted.

Statistical analysis as function of the measuring time was done on the basis of successive scans (cf. Fig. 2A and fig. S1) of a defined surface area in constant current mode with a fixed bias voltage, each acquired image of which was evaluated separately. The evaluation considered the numbers of reacted molecules of border and of center molecules *N*_B/C,R_ as well as the number of available molecules *N*_B/C,O_ + *N*_B/C,R_, which is the number of molecules in the topography that can be attributed one of the states *O* and *R*. Because of nonuniform distortion of the data, e.g., due to drift, these quantities might vary from scan to scan.

For the current-dependent measurements shown in Fig. 2E, a fully intact monolayer area was selected. In the next step, a section of 20 nm by 20 nm was scanned at a certain tunneling current of *I*_set_ = 5 pA with the bias voltage at *U*_bias_ = 2 V. After this, a larger area was scanned, including the supposedly affected area, at noninvasive tunneling parameters, i.e., in imaging mode, to evaluate the number of reacted molecules. In the next scan, again the 20 nm by 20 nm area was scanned at *U*_bias_ = 2 V, increasing the tunneling current by one order of magnitude. The described procedure was repeated until lastly a current of *I*_set_ = 5000 pA was used, and the area was studied in imaging mode each time. For the evaluation, only molecules were counted that had switched into state *R* during the last scan and were not already reacted because of one of the previous 2 V scans.

The pulsed experiments (cf. Fig. 3) were carried out as follows. First, a fully intact monolayer area was identified tunneling at a noninvasive bias voltage of *U*_bias_ = 0.4 V and a current of *I*_set_ = 50 pA at which no reaction is induced. Second, the voltage pulse of a certain height was applied as depicted in Fig. 3E. Using the feedback loop, while ramping the bias voltage up and down and while holding the maximum voltage, the tunneling current was kept constant at a predefined value of 5 pA for the first pulse. After ramping the voltage back to 0.4 V, the surface section was studied in imaging mode to evaluate the result of the pulse experiment. This procedure was repeated at the same surface area for the following 50 and 500 pA pulses using the same maximum voltage. For every voltage, a different and previously unreacted surface area is used.

Normalization of the data presented in Fig. 3D was done as follows: The histograms for bin *i* were normalized to the number of available molecules within the annulus *i* of area *A_i_* = π × [(*r_i_*)^2^ − (*r*_*i*−1_)^2^], *i* ∈ ℕ, *r*_0_ = 1 nm, *A*_0_ = π × (*r*_0_)^2^ and *r*_*i*+1_ = *r_i_* + 2 nm that leads to a distribution that decreases with increasing bin number. This is exemplarily depicted as inset of Fig. 3C for *U*_bias_ = 2.7 V, where fitting of all the distributions as exponential decay yields an average lateral decay constant of λ = 2.1 ± 0.2 nm^−1^.

The errors given in Fig. 2 (A, E, and F) result from the stochastic error for *N*_*O*/*R*_ observed events, as well as the random errors of σ(*N*_B/C,O/R_), the latter determined by repeated evaluation of the same datasets. In addition, if further processed, Gaussian error propagation was applied. The reactions can be treated as independent observations as supported by the fact that the rate equation model, which implicitly requires independent events, is found to be well describing the presented data of Fig. 2A. In detail, the random errors are determined to be as follows: for the data from Fig. 2A, σ(*N*_B/C,R_) ≈ 7%; the data from Fig. 2E, σ(*N*_B,R_) ≈ 1%; and for the data in Fig. 2F, it was found that σ(*N*_B,R_) ≈ 0.5% and σ(*N*_C,R_) ≈ 5%. The differences are attributed to variations of the tip imaging quality and the size of the scanned area evaluated. Furthermore, for the data in Fig. 2F, the difference is also due to the fact that the two reaction states of the center molecules are harder to differentiate than for the border molecules because of a smaller change in contrast as a result of the reaction. The errors for the data in Fig. 3 (F and G) are given by the stochastic errors. The errors in fig. S1 are given by the stochastic error and a random error of σ(*N*_B/C,R_) ≈ 4%.

The errors provided for every fit result represent the 68% confidence interval.

### Rate equation model

The fit of the rate equation model (Eq. 1) in Fig. 2A was done as follows: Because of randomly appearing drift, the data were not all collected at the very same surface area. As in periods of large, directed drift, the fraction of reacted molecules present in the topography is artificially reduced, and periods showing large spatial movements were excluded from analysis. Instead, positions before and after drift were treated as independent datasets. The two datasets overlapping for small fractions of reacted molecules (circles and triangles pointing down) were correlated minimizing the sum of the squared point-wise differences between both data sets. Then, the best fit with the solution of the rate equation model given in Eq. 3

$$\kappa =\frac{k_{B/C,O\to R}}{k_{B/C,O\to R}+k_{B/C,R\to O}}$$ (2)

$$N_{B/C,R}(t)=\kappa \cdot N_{B/C,\text{total}}[1-\text{exp}(-(k_{B/C,O\to R}+k_{B/C,R\to O})t)]$$ (3)

was identified, shifting the data along the time axis in this way identifying time zero. With the time of the first two datasets fixed, the fit was reapplied including the dataset of the next position allowing these to shift along the temporal axis to find the best fit. The same procedure was also applied for the last dataset. After that, each of the datasets was once again allowed to shift along the temporal axis to find the optimal correspondence between data and fit. The result of the fit thus takes into account all the collected data. The lastly used shifts are based on the best fit between model and the temporal evolution of the border molecules.

In the next step, the extracted rate constants were implemented into a simulation of the molecular grid, i.e., on a discrete and finite lattice with every lattice point representing a molecule to quantify finite size effects. The total numbers of molecules *N*_B/C, total_ were chosen in correspondence to the areas studied experimentally, and the molecules were divided into equal amounts of border and center molecules. Starting with all molecules in state *O*, for each iteration, “scan”, every single molecule was allowed to make the transition into state *R* (and if in a later scan the molecule’s state is *R* already, the reverse transition) with the probability being given by the extracted rate constants of the corresponding molecule orientation. This procedure was repeated until the predefined number of scans was reached, before starting again with a previously undisturbed molecular lattice. In this way, a desired amount of independent time traces were generated. The dotted lines shown in Fig. 2A are averaged curves of 10^5^ individual traces. The difference between each simulated data point and the analytic solution is less than 1.5% of the respective value. The areas shaded in gray depict the calculated SD environments that account for the deviation of the individual time traces from the average resulting from the finite size of the lattice paired with the stochastic nature of the simulated processes.

### Density functional theory

NEB calculations were performed using DFT as implemented in the Vienna Ab initio Simulation Package (*40*–*42*), version 5.4.4, together with the Transition State Tools for VASP (vtsttools), which adds the climbing image method (*43*) and improved tangent definition (*44*).

All DFT calculations used a plane wave basis set, the projector-augmented wave method (*45*, *46*) and the nonlocal optB86b-vdW functional (*47*) to account for dispersion interactions. A plane wave energy cutoff of 400 eV was used with a single Γ centered k-point due to the large systems studied. The electronic self-consistent loop was converged to 10^−6^ eV using no symmetry constraints (ISYM = 0) and high precision (PREC = Accurate). Gaussian smearing was used (ISMEAR = 0) for partial occupancies with the smearing width set to 0.05 eV. Because neither the Ag surface nor the *fac*-Re(bpy)(CO)_3_Cl (bpy = 2,2′-bipyridine) molecule is magnetic, nonspin-polarized calculations (ISPIN = 1) were performed. Initial relaxation of the clean Ag(001) surface was performed considering both the lattice and ionic degrees of freedom until the norms of all the forces was smaller than 0.01 eV/Å. For all relaxations of the Ag(001) surface with adsorbed molecules, only the ionic degrees of freedom were considered and the lattice parameters were kept fixed. For all relaxations, the atoms in the bottom layer of the Ag(001) surface were kept fixed.

To obtain the initial state for the NEB calculations, a single molecule was placed on a three-layer 7 × 7 × 1 Ag(001) surface and relaxed. The final state was then obtained by dissociating the Cl ion from the molecule and placing it at the closest hollow site either behind, to the side, or in front of the molecule and relaxing the system (movies S1 to S3).

The NEB calculations consisted of 10 interpolated images between the initial and final states that were relaxed using a three-stage quasi-Newton (IBRION = 1) procedure with the climbing image method (LCLIMB = .TRUE) and the spring constant between the images set to 5.0 eV/Å^2^. For each relaxation stage, the following settings were used: Stage 1 used a very low dimensionality parameter (NFREE = 2) with a rough force convergence criterion of 0.3 eV/Å. State 2 increased the dimensionality parameter to 10 and reduced the force convergence criterion to 0.1 eV/Å. Stage 3 further increased the dimensionality parameter to 20 and, lastly, reduced the force convergence criterion to 0.01 eV/Å. Bader charge analysis (*48*, *49*) was performed for each image in the NEB path shown in Fig. 4A by performing single-point calculations (NSW = 0) with LCHARG = .TRUE and LAECHG = .TRUE.

For the calculation of the Cl dissociation inside the monolayer, a three-layer 15 × 8 × 1 Ag(001) surface was used on which four layers of molecules were placed according to the experimentally measured STM pattern (see fig. S9). This is a simplified model because the experimental monolayer has very large periodicity in one direction [14.4 nm; (*33*)]. Thus, the full monolayer is too large to model with DFT, and to avoid a mismatch between the periodic images of the four rows of molecules, they were separated by roughly 6 Å in the long direction. The system [monolayer + Ag(001) surface] was then relaxed to obtain the original state *O*, after which a Cl ion from one of either the border or center molecules was dissociated and placed at the closest hollow site to the side of the reacted molecule. The system was then relaxed to obtain the reacted state *R* of the monolayer, and the dissociation energy was calculated as follows

$$E_{X,D}=E_{X,R}-E_{O},\text{with}\;X=\{B,C\}$$ (4)

Here, *E*_*X*,R_ is the total energy of the monolayer with one molecule in the reacted state and *E*_O_ is the total energy of the original monolayer. *X* = {B, C} refers to either a reacted border, B, or center, C, molecule.
