# Supplementary material for: Making and breaking of chemical bonds in single nanoconfined molecules
Source: Sci Adv. 2022 Sep 9;8(36):eabq7776. doi: 10.1126/sciadv.abq7776 (PMC9462694; doi:10.1126/sciadv.abq7776)
Supplement: Supplementary file 1 — Figs. S1 to S10 [file sciadv.abq7776_sm.pdf]

Science Advances
AAAS

Supplementary Materials for

Making and breaking of chemical bonds in single nanoconfined molecules

Ole Bunjes *et al.*

Corresponding author: Martin Wenderoth, martin.wenderoth@uni-goettingen.de

Sci. Adv. **8**, eabq7776 (2022)
DOI: 10.1126/sciadv.abq7776

The PDF file includes:

Figs. S1 to S10
Legends for movies S1 to S3

Other Supplementary Material for this manuscript includes the following:

Movies S1 to S3

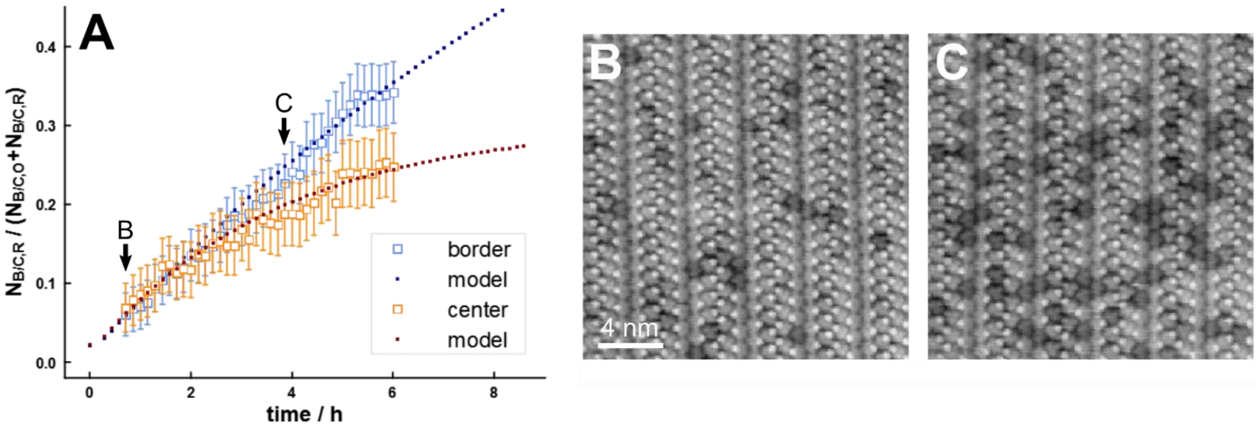

Fig. S1. Additional time series for small fractions of reacted molecules. (A) $N_R(t)$ normalized to the number of molecules available in the scanned area, as evaluated separately for both orientations of molecules, at constant tunneling parameters of U_{bias} = 1.875 V and I_{set} = 500 pA, scan area ca. 20 × 20 nm², duration of one scan t_{scan} = 515 ± 5 s. The dashed lines show the result of the rate equation model. The data were shifted along the horizontal axis for minimizing the correlation coefficient R^2 for the border molecules, cf. 'Methods'. The black arrows indicate the data points that stem from the analysis of the two topographies (B) and (C). Effective rate constants: $k_{B,O\rightarrow R} = 4.3 \pm 0.2 \cdot 10^{-3}\ s^{-1}$, $k_{B,R\rightarrow O} < 10^{-10}\ s^{-1}$, $k_{C,O\rightarrow R} = 4.9 \pm 0.4 \cdot 10^{-3}\ s^{-1}$, $k_{C,R\rightarrow O} = 11.2 \pm 0.9 \cdot 10^{-3}\ s^{-1}$.

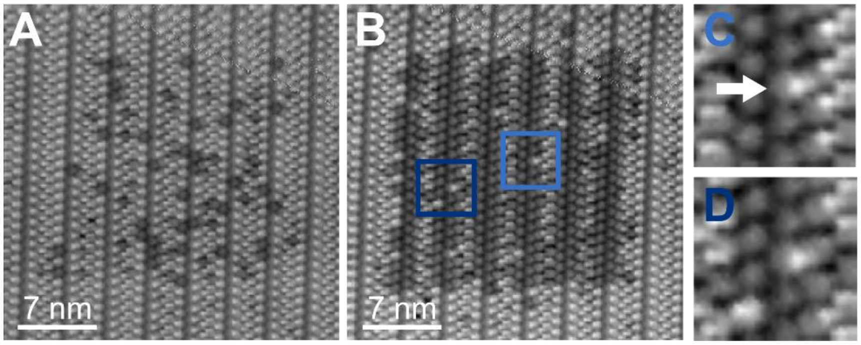

Fig. S2: Center molecules show also reverse reaction. (A) Scan in imaging mode (U_{bias} = 1 V and I_{set} = 50 pA) after excitation scan (U_{bias} = 2.9 V and I_{set} = 5 pA). (B) Scan in imaging mode (U_{bias} = 1 V and I_{set} = 50 pA) after a second excitation scan (U_{bias} = 3.2 V and I_{set} = 5 pA). The molecule framed in light blue and indicated by the white arrow in (C) has undergone the reverse reaction. For easier comparison framed in dark blue and shown in (D) there is a border molecule that has not undergone the transition into state *R*. (C+D) Area sizes circa 4 × 4 nm^2.

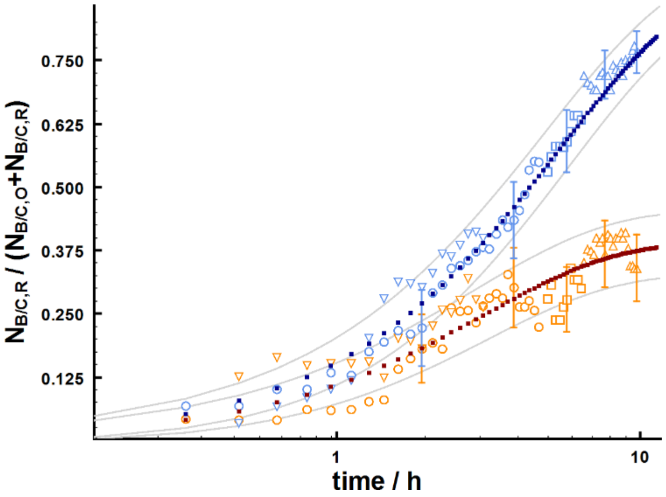

Fig. S3: Data from Fig. 2(A) in a semilog-plot as comparison for the data in Fig. 3(F). As the data are recorded in constant current mode, the time is proportional to the electron dose injected into the molecule-surface system. $N_{B/C,R}(t)$ normalized to the number of molecules available in the scanned area, as evaluated separately for both orientations of molecules (border = blue, center = orange), at constant tunneling parameters of U_{bias} = 2 V and I_{set} = 50 pA, scan area ca. 10 × 10 nm², duration of one scan t_{scan} = 572 ± 4 s. Each type of symbol represents a different data set. The dotted lines show the result of the rate equation model and the gray lines represent the corresponding standard deviation, details on both in 'Methods'.

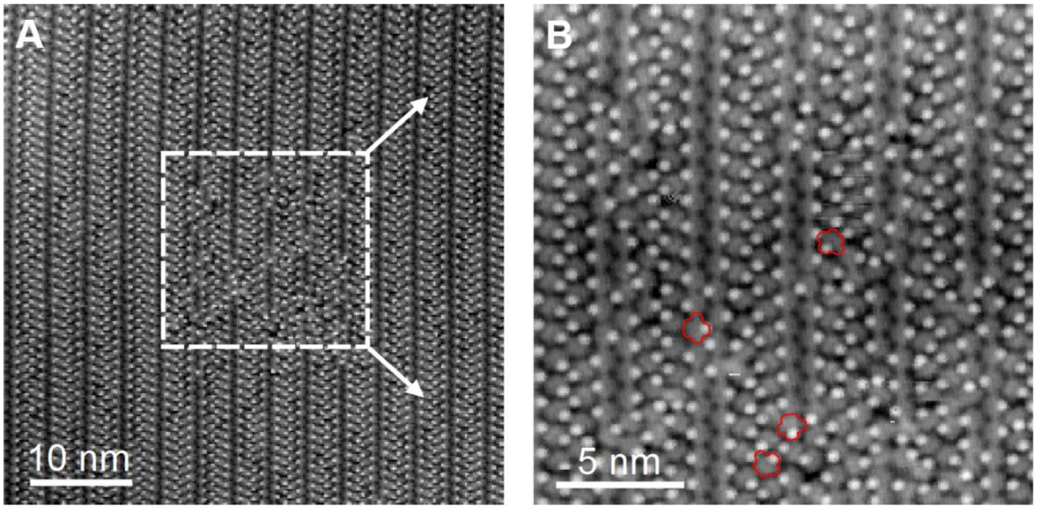

Fig. S4: Reaction is not induced due to mechanical tip-molecule contact. (A) Topography ($U_{\text{bias}} = 1$ V, $I_{\text{set}} = 50$ pA) after the region framed has been scanned at $U_{\text{bias}} = 10$ mV and $I_{\text{set}} = 500$ pA. (B) Region framed in (A) showing disruption of the molecular structure that might be interpreted as rotation of the complexes induced by a repulsive tip-molecule interaction at small tip-sample distances, without causing a chemical reaction similar to the one discussed in the main text. Exemplarily, four complexes showing a different configuration than their neighboring equals are highlighted in red.

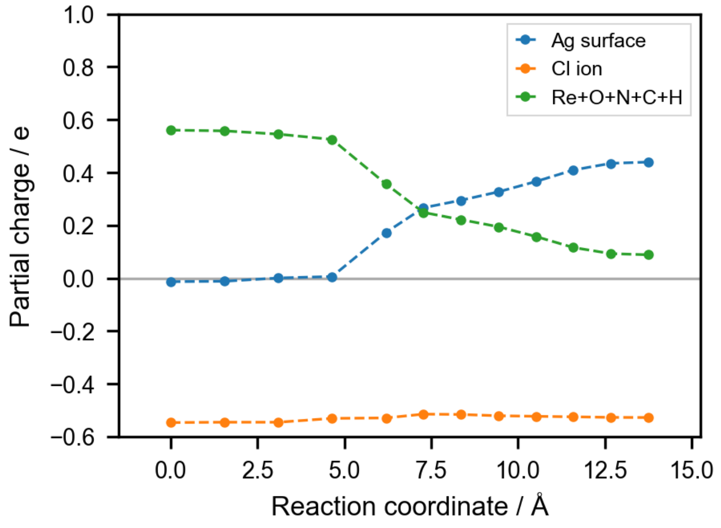

Fig. S5: Bader charge analysis for the NEB path in Fig. 4(A) (and Fig. S6(B)). The blue dots are the sum of the partial charges for all Ag atoms in the surface. Orange dots denote the partial charges for the Cl ion and the green dots are the sum on the partial charges for all Re, O, N, C and H atoms. The dashed lines are guides to the eye.

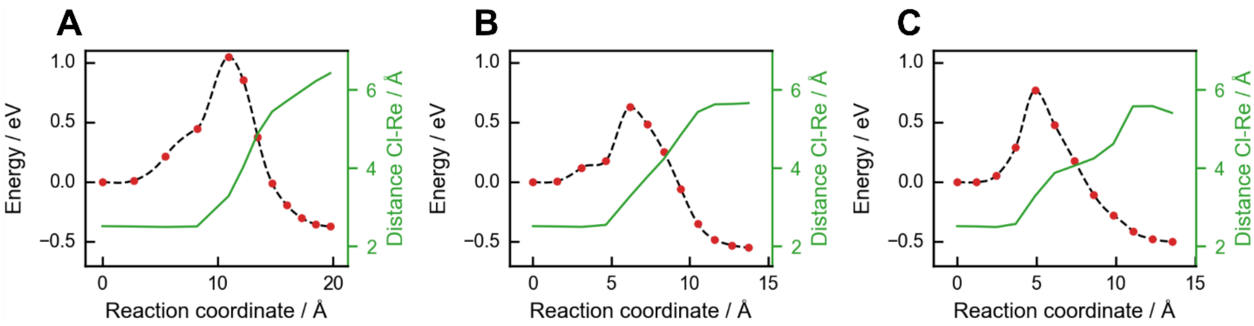

Fig. S6: Minimum energy pathways for Cl ion dissociation obtained via density functional theory based nudged elastic band calculations (NEB). (A) Is the energy barrier for dissociation in the backwards direction (see Movie S1), (B) for the sideways direction (see Movie S2) and (C) for the forward direction (see Movie S3). Note that shown in (B) are the same NEB calculations as shown in Fig. 4(A). For each minimum energy pathway, the distance between the chloride ion and the rhenium center is shown in green.

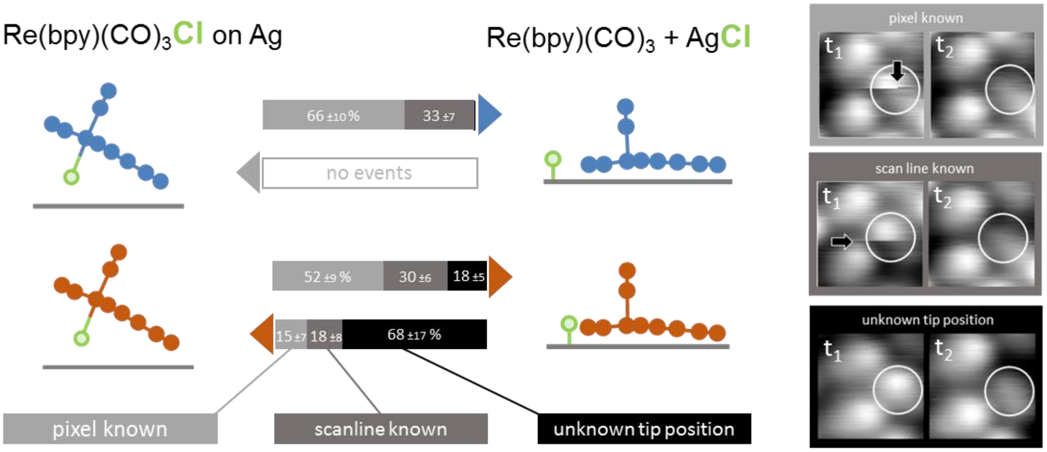

Fig. S7: Spatial analysis of the reaction at 1.875 V and at 2 V. Separately for border (blue) and center molecules (orange), it is evaluated with which relative frequency the transitions from state *O* (intact adsorbed molecule, left) to *R* (dissociated molecule, right) and the reverse reaction are observed as a sharp contrast (pixel or scan line known) in the STM topographies. The chloride ion is depicted in green. The errors are given by the statistical errors based on the number of reactions considered. In the topographies at time t_1 the encircled center molecule is initially in state *O*. The tip position at which the reaction is visible in the topography is indicated by the black arrow. In the consecutive scan, at time t_2, the encircled molecule is found in state *R*.

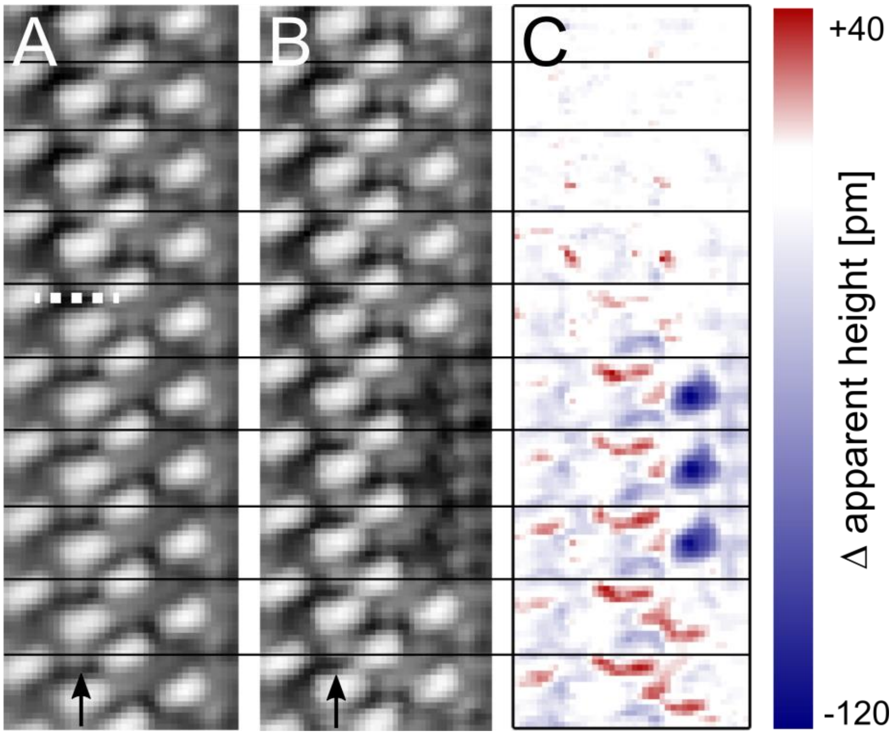

Fig. S8: Dissociation reaction induces relatively long-range structural disruption into molecular pattern, i.e., the induced structural change reaches beyond the direct neighbors of the dissociating molecules. (A) Reference topography, before pulsed experiment. The column of center molecules indicated by the black arrow does show two different configurations that are separated by the white dotted line. (B) Topography after some of the border molecules have undergone the transition into the reacted state. The column of center molecules indicated by the black arrow does show only one configuration similar to the one of the topmost molecules in the same column in (A). Please note that due to the dissociation of some molecules also the local density of states (contributing to the constant current topographies) is expected to change. The change found for the column indicated by the black arrow, however, is likely to originate from a structural rearrangement of the molecules, since a very similar structure is already present in the upper part of the column indicated by the black arrow in the reference topography, in which all the molecules are intact. Same data set as shown in Fig. 3A. (C) Difference Δ in apparent topographic height = (A) minus (B). Note the asymmetric color scale used to highlight the induced differences. Tunneling parameters U_{bias} = 0.4 V and I_{set} = 50 pA, area size 3.2 × 10 nm².

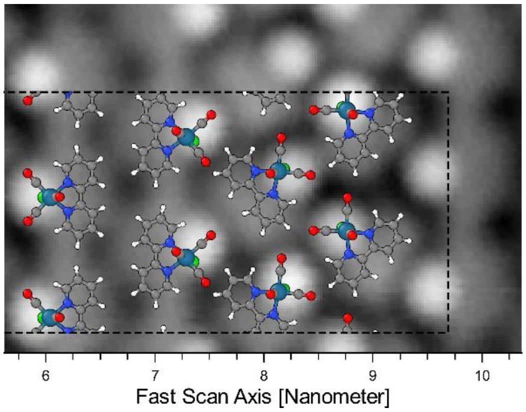

Fig. S9: The relaxed molecular arrangement used to model the monolayer. Where the Cl ion of each molecule is centered at the closest hollow site of the Ag(001) surface. Note that the molecules are to scale, and the dashed line denotes the unit cell used in the DFT calculations. Note that due to the very large periodicity of the experimental pattern (14.4 nm) only 4 layers of molecules were used, separated by vacuum in the periodic direction (along the fast scan axis in the figure).

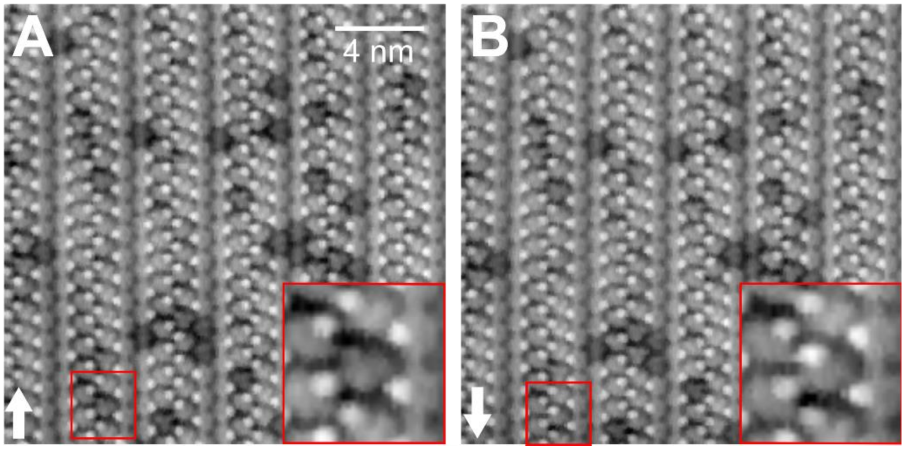

Fig. S10: Reverse reaction is independent of state of surrounding molecules.
(A) Topography showing several molecules during an excitation scan (U_{bias} = 1.875 V and I_{set} = 500 pA). Molecule in the center of the region framed in red is first in state *R* and in the next topography (B) back in its state *O*. The surrounding molecules, its direct neighbors, are all in state *O*. The inset shows a magnification of the region framed in red, area size circa 2.5 × 2.5 nm². The white arrows indicate the scan direction along the slow scan axis.

Movie S1.
Dissociation in the backwards direction, corresponding to NEB calculations presented in Fig. S6(A).

Movie S2.
Dissociation in the sideways direction, corresponding to NEB calculations presented in Figs. 4(A) and S6(B).

Movie S3.
Dissociation in the forward direction, corresponding to NEB calculations presented in Fig. S6(C).
